# Kinematic markers of skill in first-person shooter video games

**DOI:** 10.1093/pnasnexus/pgad249

**Published:** 2023-07-31

**Authors:** Matthew Warburton, Carlo Campagnoli, Mark Mon-Williams, Faisal Mushtaq, J Ryan Morehead

**Affiliations:** School of Psychology, University of Leeds, Leeds, West Yorkshire, LS2 9JT, UK; School of Psychology, University of Leeds, Leeds, West Yorkshire, LS2 9JT, UK; School of Psychology, University of Leeds, Leeds, West Yorkshire, LS2 9JT, UK; Bradford Institute for Health Research, Bradford Hospitals National Health Service Trust, Bradford, BD9 6RJ, UK; National Centre for Optics, Vision and Eye Care, University of South-Eastern Norway, Kongsberg 3616, Viken, Norway; School of Psychology, University of Leeds, Leeds, West Yorkshire, LS2 9JT, UK; Centre for Immersive Technologies, University of Leeds, Leeds, West Yorkshire, LS2 9JT, UK; School of Psychology, University of Leeds, Leeds, West Yorkshire, LS2 9JT, UK; Centre for Immersive Technologies, University of Leeds, Leeds, West Yorkshire, LS2 9JT, UK

**Keywords:** kinematics, sensorimotor, skill, assessment, video games

## Abstract

Video games present a unique opportunity to study motor skill. First-person shooter (FPS) games have particular utility because they require visually guided hand movements that are similar to widely studied planar reaching tasks. However, there is a need to ensure the tasks are equivalent if FPS games are to yield their potential as a powerful scientific tool for investigating sensorimotor control. Specifically, research is needed to ensure that differences in visual feedback of a movement do not affect motor learning between the two contexts. In traditional tasks, a movement will translate a cursor across a static background, whereas FPS games use movements to pan and tilt the view of the environment. To this end, we designed an online experiment where participants used their mouse or trackpad to shoot targets in both visual contexts. Kinematic analysis showed player movements were nearly identical between contexts, with highly correlated spatial and temporal metrics. This similarity suggests a shared internal model based on comparing predicted and observed displacement vectors rather than primary sensory feedback. A second experiment, modeled on FPS-style aim-trainer games, found movements exhibited classic invariant features described within the sensorimotor literature. We found the spatial metrics tested were significant predictors of overall task performance. More broadly, these results show that FPS games offer a novel, engaging, and compelling environment to study sensorimotor skill, providing the same precise kinematic metrics as traditional planar reaching tasks.

**Significance Statement**

Sensorimotor control underpins human behavior and is a predictor of education, health, and socioemotional well-being. First-person shooter (FPS) games hold promise for studying sensorimotor control at scale, but the visual feedback provided differs from traditional laboratory tasks. There is a need to ensure they provide measures that relate to traditional tasks. We designed an experiment where the visual contingency of movements could be varied while participants shot targets. Participant's movements were similar between contexts, suggesting the use of a common internal model despite the sensory differences. A second experiment observed canonical learning patterns with practice and found measures of mouse control strongly predicted overall performance. Our results highlight the opportunity offered by FPS games to study situated skilled behavior.

## Introduction

Video games are an increasingly ubiquitous form of entertainment, with many gamers so skilled they are employed to play in tournaments where hundreds of millions of dollars are awarded every year (1). Action video games (encompassing first-person shooter [FPS] games) are especially popular and typically engage a range of cognitive, perceptual, and motor abilities (2, 3). The links between gaming and perceptual and cognitive abilities have been well studied, finding that action video game players show better performance among many faculties (2, 4). Few studies, however, have focused on skill development in these games and, in particular, the role of the motor system in reaching and maintaining good performance, despite its clear relevance (5, 6). This is particularly disappointing given the importance of sensorimotor control and learning in academic attainment (7), neurological deficit (8), and socioemotional development (9).

The possibility of using gameplay to measure skill development has long been recognized (10, 11), allowing the full history of a player's trajectory to be tracked in an automatic and naturalistic manner (12). Previous research tends to measure skill development in games using in-game measures, such as a player's score per game (12), proprietary skill metrics (13–15), or multiplayer metrics like the ratio of kills and assists to deaths (16). While these provide interesting examples of how skills broadly improve, they do not inform what specific subcomponents of behavior contribute to motor development. For example, an equal improvement in score may arise because of changes in decision-making or motor execution.

Two recent studies have used a commercial FPS aim-trainer to characterize motor performance by isolating the skill of aiming at stationary targets (17, 18). In FPS games, players see the virtual world from the first-person perspective of their character and use their mouse to control the character's view, typically to aim toward and shoot enemies. The studies have found that broad measures of motor performance, such as hit accuracy and hit rate, improve with practice (18) and that specific features of a movement, such as reaction time and precision of movements, correlate well with a derived measure of motor skill (17). Similar tasks have been embedded into commercial FPS games, finding highly skilled gamers were better on a range of temporal measures (19, 20), and actual gameplay data have been analyzed, finding professional players have improved hit rates, lower reaction times, and more efficient movements, compared with amateurs (21).

While the study of skill in FPS games has only recently come to the fore, the act of moving toward a target has been studied extensively in a laboratory setting using visually guided reaching tasks. Much of our modern understanding of motor control and learning comes from studies where participants interact with a digitizing tablet or a robotic arm, representing their movements as those of a cursor moving across a screen, with many recent studies being performed using computer mice or trackpads (22–25). Further, the kinematic analysis of computer mouse movements has been common in the field of human–computer interaction (26–29).

Traditional computational schema for movement execution has an inverse model that translates an initial and desired state into a motor command (30, 31). More recent optimal feedback control frameworks use a control policy that generates motor commands through modification of feedback gains to achieve task goals (32, 33). Most computational frameworks propagate a copy of the outgoing motor through a forward model to predict the sensory consequences of the movement and integrate it with observed sensory feedback to compensate for noise and inherent sensorimotor loop delays (34, 35), with this optimal state estimate fed to the control policy to give closed-loop feedback. Many of these operations are thought to be carried out by distinct neural substrates (though this is an oversimplification, see 36): feedback gains within the premotor and primary motor cortices (37), forward modeling within the cerebellum (38), and state estimation within the parietal cortex (39).

Current computational models do not exactly specify the nature of the sensory predictions they employ. If sensory predictions are generated and compared at the level of primary sensory feedback, distinct forward models would be required to make accurate feedback corrections in traditional laboratory and FPS-style tasks, given the large differences in visual motion that result from a movement. Movements of an arm or computer mouse in a typical experiment translate a cursor across a static environment (known in human–computer interaction as Pointing, henceforth *Point*) in much the same way someone would interact with their computer's desktop. In contrast, FPS games use the movement of the mouse to pan and tilt the view of the game while the cursor remains central to the screen, bringing peripheral targets to the cursor (known in the gaming industry as Mouselook, henceforth *Look*). It is therefore possible the feedback differences between contexts could cause people to move in observably different ways. One study did find that acquiring targets when Looking took longer than Pointing (40), but movement requirements were not properly equated between contexts. No other work has compared movements between these contexts.

To investigate whether the visual differences between Looking and Pointing lead to observable differences in motor behavior, we designed a simple task where players had to execute movements in both contexts. Participants attempted to make center-out reach movements to land on and click a target to “pop” it while under imposed time pressure. Spatial and temporal properties of the movements executed in both contexts showed high correlation, with only slightly elevated reaction and correction times in Looking movements. To further explore behavior in FPS games, we ran a second experiment, modeled on popular aim-trainer games, that had participants complete 20 rounds of Looking movements consisting of shots to a sequence of 48 targets. Here, we observed a range of classic observations from the reaching literature and related the spatial measures of FPS aiming skill to overall performance.

## Results

### Experiment 1

**Fig. 1.**
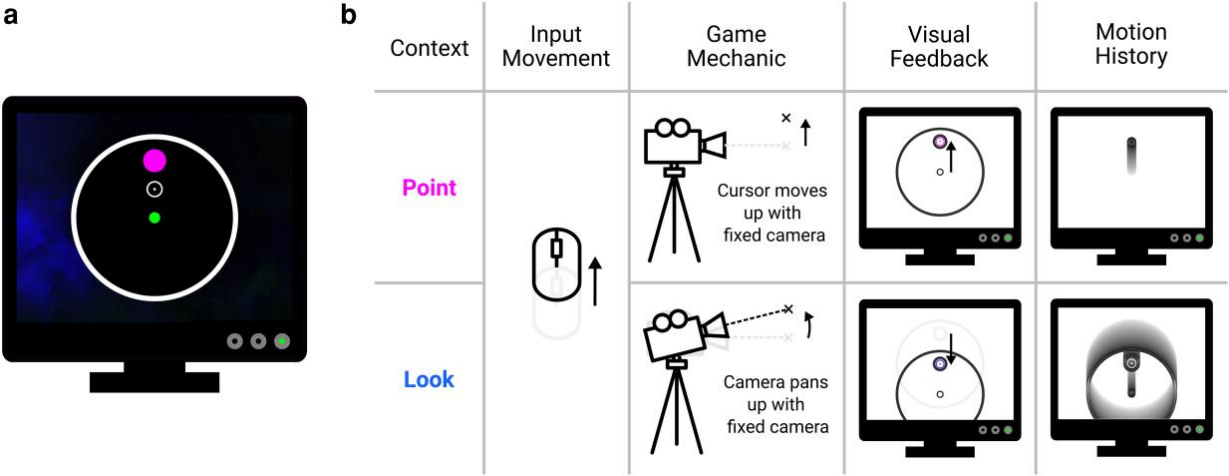
Center-out reaching paradigm. a) Participants clicked on a start point located in a circular target plane. Upon a target appearing, participants attempted to use their mouse or trackpad to move a cursor to the target and left-click to shoot it. b) Participants could control the cursor in two contexts. In the Point context, participants’ mouse movements translated the cursor across an otherwise static scene. In the Look context, participants’ mouse movements panned a camera while the cursor remained central to the camera's view (typical of FPS-style games), giving the visual effect that everything except the cursor moves. While a common input movement can reach the target in either context, the motion history shows how this difference in feedback leads to different progressions of visual feedback over time.

In experiment 1, participants (*n* = 50) used the mouse or trackpad of their personal computer to perform a center-out reach task. Upon clicking a start point, they had to move to and click a target within a time limit to shoot it; otherwise, the target would disappear (Fig. 1a). A staircasing procedure was used to manipulate the time limit throughout a block, where the time limit of a pair of interleaved staircases increased or decreased by 30 ms in response to an unsuccessful or successful trial, respectively, giving participants personalized time pressure that resulted in success on roughly 50% of the trials. Participants completed a block of 320 trials in both the Point and Look contexts, where mouse movements either translated the cursor across a static background or panned and tilted the game's view while the cursor remained static respectively (Fig. 1b; see Video S1 for a demonstration). The visuomotor contexts were equated so that each required nearly identical mouse movements to shoot a given target, allowing direct comparisons.

### Participants required more time to shoot targets in the Look context

**Fig. 2.**
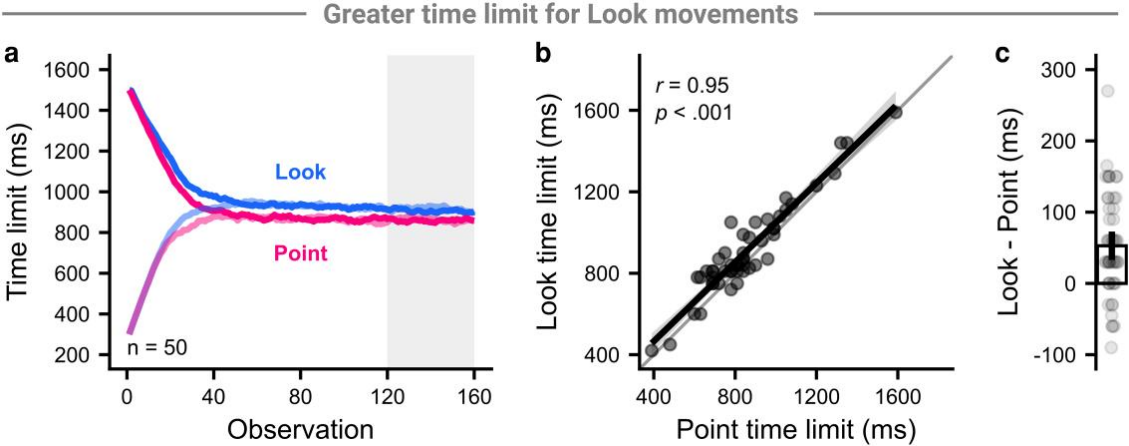
Comparison of trial time limit between contexts. a) Solid lines show the mean time limit across participants per context and staircase, starting either high (darker) or low (lighter). The last 40 trials are highlighted as comparisons between metrics were made over the last 40 trials of each context's staircases through the rest of this experiment. b) Points show the within-subject median time limit per context, the thick line shows the regression line, and the shaded region shows the 95% CI of the regression line. c) Points show participant differences between the contexts, and the bar and vertical line show the group mean and 95% CI of the difference, respectively.

Staircase time limits progressed similarly between contexts, appearing to become roughly asymptotic by the end of the block (Fig. 2a). The median time limit over the last 40 trials of each staircase showed no significant difference for the Point (*t*(49) = −0.78, *P* = 0.437, *d* = −0.11) or Look context (*t*(49) = −1.14, *P* = 0.259, *d* = −0.16), so we calculated a single asymptotic time limit per context over the last 40 trials of both staircases combined and used this as our measure of participant skill in this experiment. Asymptotic time limits per participant were highly correlated between contexts (*r*(48) = 0.95, *P* < 0.001; Fig. 2b) and were longer in the Look context (mean difference [95% CI] = 53 ms [33–73 ms], *t*(49) = 5.18, *P* < 0.001, *d* = 0.73; Fig. 2c). While participants using a mouse had lower asymptotic time limits than those using a trackpad when averaged over context (mouse: 794 ms, trackpad: 1,037 ms, *t*(48) = −4.46, *P* < 0.001, *d* = −1.26), the between-context correlation (Fisher's *z* = −0.18, *P* = 0.856) and difference (*t*(48) = −1.71, *P* = 0.093, *d* = −0.48) was not significantly different. Further, the between-context difference was not significantly different based on the number of hours playing games per week (*F*(4, 45) = 0.87, *P* = 0.489, $\eta_{G}^{2}$ = 0.07) or whether participants played FPS games (*t*(48) = 1.34, *P* = 0.188, *d* = −0.44).

### Spatial measures are identical between contexts

**Fig. 3.**
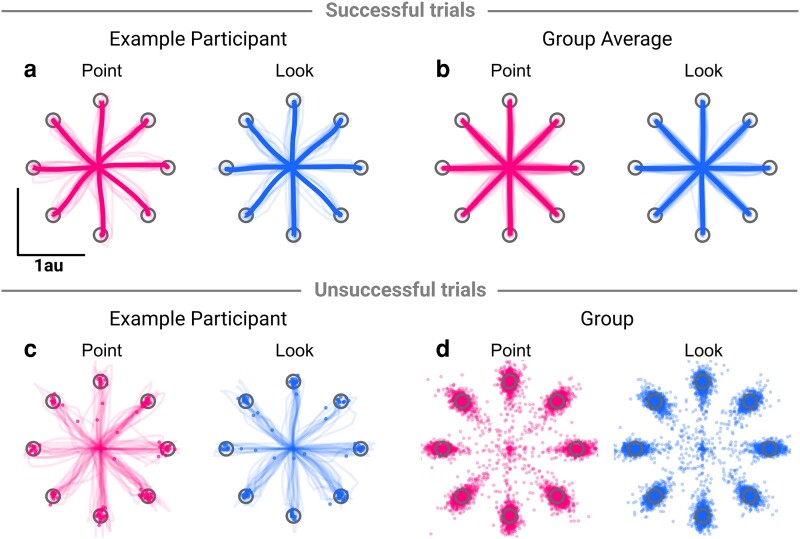
Hand paths for successful trials a, b) and endpoint distribution for unsuccessful trials c, d). a) Hand paths for an example participant on successful trials, both for individual movements (thin lines) and for average movements to each target (thick lines). b) Average hand paths for each target on successful trials, both for participants (thin lines) and for group averages (thick lines). c) Hand paths and endpoints for an example participant on unsuccessful trials. Each thin line and point shows an individual trial. d) Endpoint distribution for all participants on unsuccessful trials.

We used the data within movements to better understand how this time difference arose. On successful trials, participant movements showed variable curvature around the ideal movement path, but average hand paths were reasonably straight and directed to the targets (Fig. 3a). Across all participants, average hand paths per target showed little curvature (Fig. 3b). While all successful trials end in the target, there were a range of ways a participant could fail a trial. The most common failure (48%) was that participants did not click in time to shoot the target, despite being inside it (Fig. 3c and d), but participants also undershot or overshot the target or missed it due to poor directional aim. There appears to be little difference in the hand paths and endpoint distribution of between contexts.

**Fig. 4.**
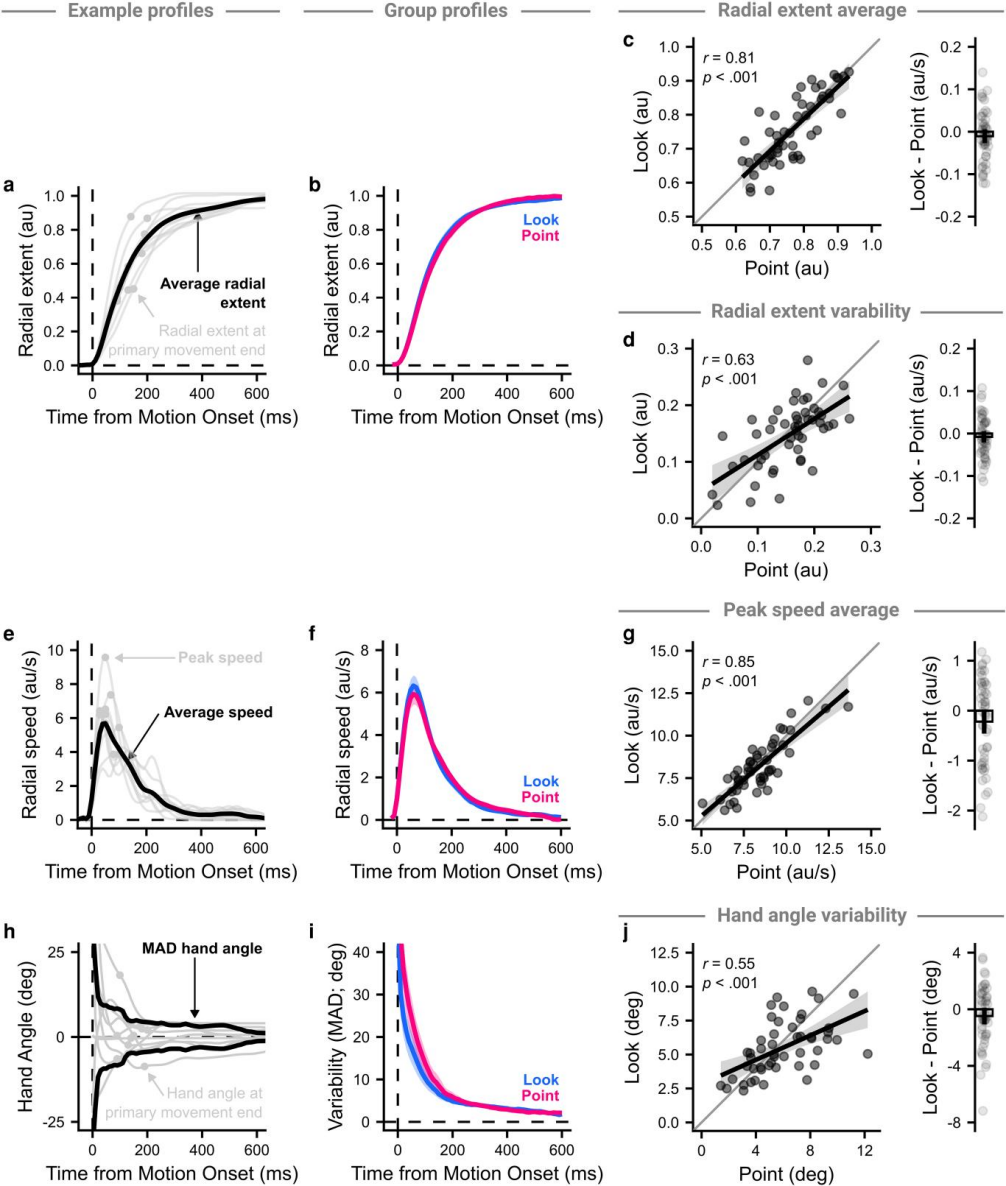
Comparison between contexts on successful trials for derived spatial measures. a) An example of 10 trials showing how the radial extent measures were extracted. The light lines show the radial extent across time of individual trials, with a point showing the radial extent when the primary movement ended, and the dark line shows the average radial extent over the individual trials. b) The lines show the average across participants per context, with the shaded regions showing 95% CI. c) The average radial extent at the end of the primary movement was compared between contexts. Left panel: Points show individual participants’ average radial extent, the thick line shows the regression fit, and the shaded region shows the 95% CI. Right panel: Within-subject difference in average extent between contexts. Points show participant differences, the bar shows the mean difference and the vertical line shows the 95% CI. d) as c) but with variability in radial extent. e–g) and h–j) as a–c) but showing how the radial speed and hand angle were compared between contexts, respectively.

Finding no apparent difference in hand paths, we investigated four spatial measures that may be able to explain the difference in time limit between contexts, which were all measured before discrete feedback corrections are thought to occur (41). Because it was possible for some movement features to be missing on unsuccessful trials, we only analyzed successful movements from the last 40 trials of either staircase to ensure a consistent sample across measures. We assessed both the radial extent over the whole movement and at the end of the primary movement (Fig. 4a). The average radial extent over the whole profile showed little difference between contexts (Fig. 4b), and the average radial extent at the end of the primary movement was strongly correlated between contexts (*r*(48) = 0.81, *P* < 0.001), with no significant difference between the Look and Point contexts (−0.01 arbitrary Unity units [au] [−0.03–0.01 au], *t*(49) = −1.27, *P* = 0.211, *d* = −0.18; Fig. 4c). Further, the variability in the radial extent of the primary movement also correlated well between contexts (*r*(48) = 0.63, *P* < 0.001), with no significant context difference (−0.01 au [−0.02–0.01 au], *t*(49) = −1.15, *P* = 0.255, *d* = −0.16; Fig. 4d).

A similar analysis was performed for reach speeds (Fig. 4e). The average speed profiles for each context were almost identical (Fig. 4f), with high correlation (*r*(48) = 0.85, *P* < 0.001), and no significant difference in average peak speed (−0.22 au/s [−0.46–0.02 au/s], *t*(49) = −1.79, *P* = 0.080, *d* = −0.25; Fig. 4g). Finally, the variability in angle from the target (Fig. 4h) was again very similar across the contexts (Fig. 4i), with high correlation (*r*(48) = 0.55, *P* < 0.001), and no significant difference in variability in hand angle at the end of the primary movement (−0.48° [−1.07° to 0.11°], *t*(49) = −1.58, *P* = 0.120, *d* = −0.22; Fig. 4j). None of the correlation coefficients or differences were significantly different between input device (*P*'s > 0.0826).

While these spatial measures are unlikely to account for the difference in time limit observed between contexts, given the high similarity, they may be able to account for good performance overall. All spatial measures were significantly correlated with asymptotic time limit when collapsed across context: average (*r*(48) = −0.63, *P* < 0.001) and variability in radial extent of the primary movement (*r*(48) = 0.50, *P* < 0.001), peak speed (*r*(48) = −0.30, *P* = 0.032), and variability in hand angle at the end of the primary movement (*r*(48) = 0.41, *P* = 0.004).

### Temporal measures reveal difference between contexts

**Fig. 5.**
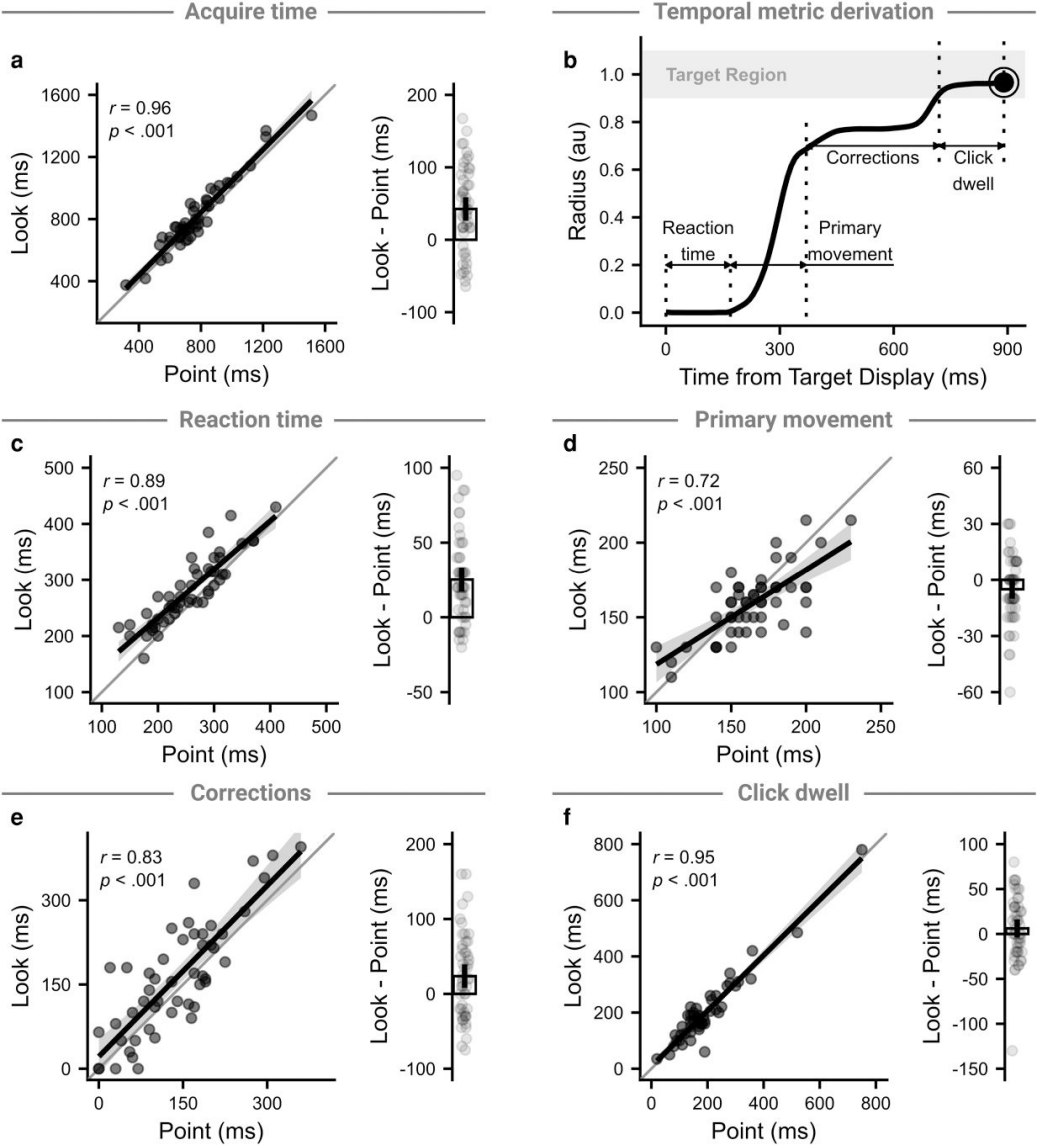
Temporal measures. a) Left panel shows the correlation between acquire time in the Look and Point contexts, with points showing participant medians, thick line showing the regression line, and the shaded region showing 95% CI for the regression line. Right panel shows the difference between the Look and Point contexts, with points showing the within-subject difference, the bar showing the group mean difference, and the vertical line showing the 95% CI in the mean difference. b) Derivation of the temporal metrics. Reaction time: time from stimuli onset to movement onset. Time to peak speed: time from movement onset to peak speed being reached. Online corrections: time from peak speed being reached to the target being entered. Click dwell: time from the target being entered to a successful shot. c–f) as a) but for the derived measures.

Because the spatial measures were all measured after motion onset and prior to corrections, other phases of the movement should account for the context difference. For successful trials, we can measure the acquire time – the total time required to go from seeing a target to shooting it. Acquire time was highly correlated between contexts (*r*(48) = 0.96, *P* < 0.001; Fig. 5a), with a higher average acquire time in the Look context (43 ms [26–59 ms], *t*(49) = 5.13, *P* < 0.001, *d* = 0.72). Given that the acquire time participants can achieve while being successful determines the asymptotic time limit, it is no surprise that the acquire time and asymptotic time limit were nearly perfectly correlated (*r*(48) = 0.99, *P* < 0.001).

Using movement kinematics, we can split the acquire time into distinct phases (Fig. 5b). We first extracted reaction time, measured as the time from a target being shown to the first sample where the radial speed was above a threshold of 0.5 au/s. We also extracted the primary movement time, between movement initiation and the end of the primary movement; the click dwell time, between the target being entered and a successful click being registered; and the correction time, between the primary movement ending and the target being entered. Again, we only use the successful trials from the last 40 observations per staircase.

Across all temporal measures, performance in the Look and Point contexts was highly correlated (reaction time: *r*(48) = 0.89, *P* < 0.001; primary movement: *r*(48) = 0.72, *P* < 0.001; corrections: *r*(48) = 0.83, *P* < 0.001; click dwell: *r*(48) = 0.95, *P* < 0.001; Fig. 5c–f). Given that these measures combine to give the total acquire time and hence interact with the asymptotic time limit, they should be able to account for the differences between the contexts. Reaction time (25 ms [17–33 ms], *t*(49) = 6.24, *P* < 0.001, *d* = 0.88) and correction time were significantly greater in the Look context (24 ms [8–39 ms], *t*(49) = 2.95, *P* = 0.005, *d* = 0.41), but neither the primary movement (−5 ms [−10–1 ms], *t*(49) = −1.93, *P* = 0.059, *d* = −0.27) nor click dwell time (6 ms [−4–16 ms], *t*(49) = 1.20, *P* = 0.235, *d* = 0.17) significantly differed between contexts (*note that the sum of differences here does not equal the difference in acquire time, as the sum of medians is not equal to the median of sums*). None of the correlation coefficients or between-context differences significantly differed between input device (*P*'s > 0.083) beside correction time (*t*(48) = −2.41, *P* = 0.020, *d* = −0.68), where only trackpad users saw a between-context difference (mouse: *t*(25) = 0.62, *P* = 0.542, *d* = 0.12; trackpad: *t*(23) = 3.44, *P* = 0.002, *d* = 0.70). When trials were averaged across both contexts, all measures except for primary movement time were highly correlated with the asymptotic time limit (reaction time: *r*(48) = 0.63, *P* < 0.001; primary movement: *r*(48) = −0.09, *P* = 0.525; online corrections: *r*(48) = 0.76, *P* < 0.001; click dwell: *r*(48) = 0.87, *P* < 0.001).

### Experiment 2

Experiment 1 showed that kinematic analysis can be successfully applied to FPS-style movements, with task performance decomposed into spatial and temporal metrics that showed high correlations between the Point and Look contexts. Further, all spatial measures correlated with the asymptotic time limit, suggesting they may be important predictors of FPS skill. However, given that this task was designed as a comparison between traditional Pointing tasks and FPS-style mouse Looking, it did not assess kinematic markers of FPS gaming skill within their typical context. We therefore designed a second experiment that only assessed Looking movements in an aim-trainer style task.

In this experiment, participants (*n* = 86) completed 20 rounds, each consisting of shots to 48 targets. To start a round, participants clicked a start point, which made a target appear as a filled circle. The next target location was also simultaneously shown as a hollow, faded circle. For the rest of the round, any time the current target was shot, the next target immediately filled in, and the following target appeared as another faded hollow circle. There was no time limit for movements, but participants were told to complete each round as quickly as they could, with a clock shown during and after the round indicating how long they had taken. The target locations were chosen as a combination of three movement distances (0.4, 0.6, and 0.8 au) and eight directions (0–315° in 45° increments), with each combination sampled twice per round. Video S2 shows a demonstration of the task.

### Looking movements show classic reaching observations

To further investigate the similarity between Pointing and Looking movements, we considered whether the Looking movements here showed classic features of reaching movements from the literature, delivered with arm reaches and shown with either actual feedback of one's hand or with Pointing style feedback. All results are consistent across mouse and trackpad users.

### Reaches are straight

**Fig. 6.**
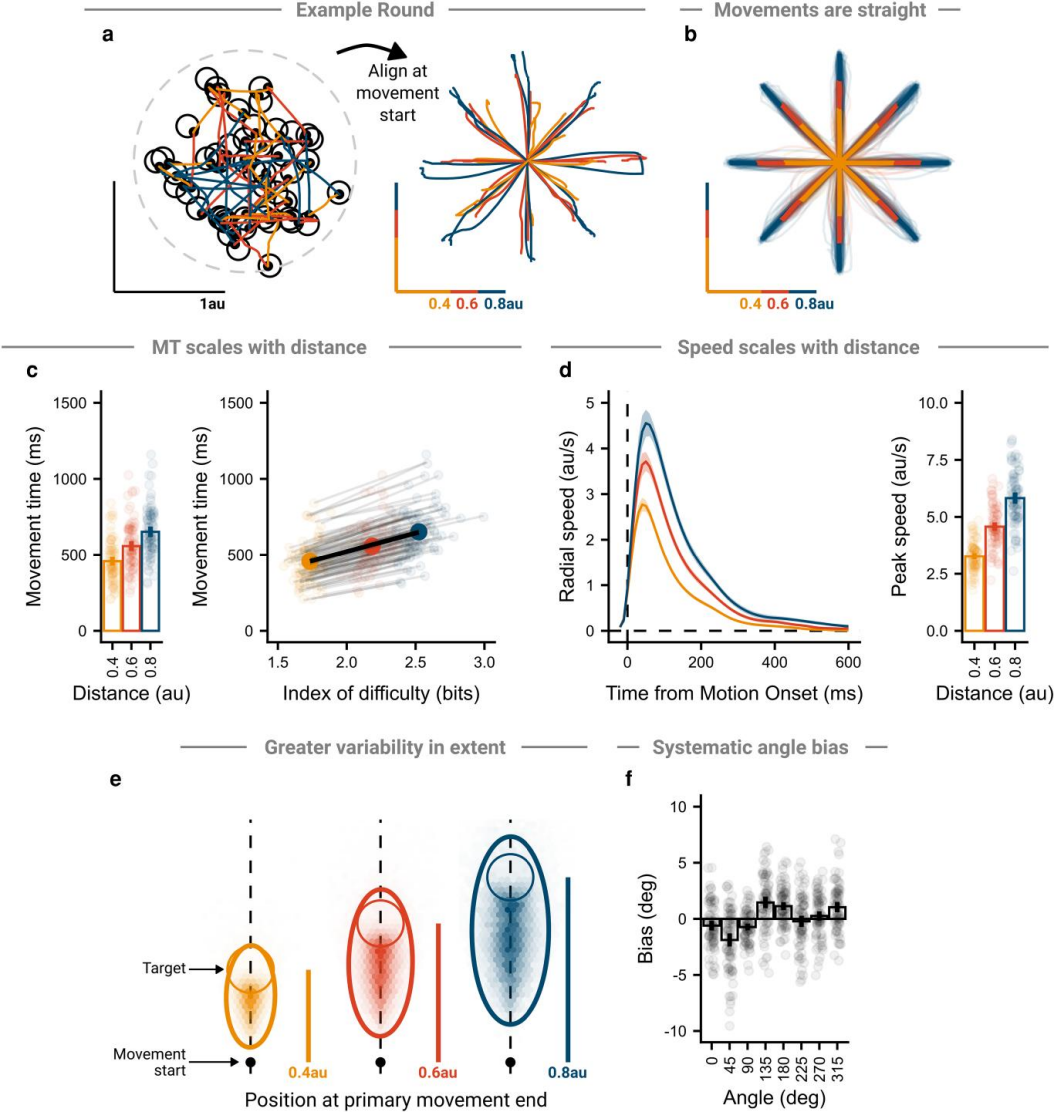
Looking movements show common features of reaching movements. a) Participants completed rounds that consisted of 48 back-to-back movements. While targets had the appearance of being randomly located, they were organized so that all combinations of three distances (0.4, 0.6, and 0.8 au) and eight angles (0°–315° in 45° increments) were tested twice. Arranging movements at their start points allowed them to be analyzed as center-out reach style movements. b) Participant movements were generally straight. Thin lines show participant average movements to each of the three distances and eight directions, with thick lines showing group averages. c) Movement times (from motion onset to successful click) scaled with the target distance. Left panel—points show participant average movement times, bars show group average, and lines show group 95% CI. Right panel—linear relationship between movement time and index of difficulty, with small points and lines showing participant average and regression slope, and large points and line showing group average and regression slope. d) Speed scaled with movement distance. Left panel—line shows group average speed profile and shaded region shows 95% CI. Right panel—points show participant average movement times, bars show group average, and lines show group 95% CI. e) Movements showed more variability in extent than direction. Heat map of cursor position at primary movement end, after aligning all movements as if directed to the target directly up. Target shown as a thin circle and 95% error ellipse shown as thick ellipse. f) Primary movements showed a systematic directional bias. Points show participant average directional error, bars show group average, and lines show group 95% CI.

By arranging movements at their initial position, we can see that, both at the individual movement level (Fig. 6a) and at the group level (Fig. 6b), movements are generally straight and aimed directly at the target (42, 43). The linearity index was calculated for the group, giving an average value of 0.08, similar in magnitude to other observations of horizontal reaching movements (44, 45). This observation is important, as it has been demonstrated that people optimize movements to ensure visual feedback cursors move in a straight line over other factors like metabolic costs (46).

### Movement time scales with distance

Movement times were found to increase as the required movement distance increased (Fig. 6c, left panel) (47, 48). A repeated measures ANOVA on participant median movement times found a significant main effect of movement distance (*F*(1.33, 112.84) = 825.37, *P* < 0.001, $\eta_{G}^{2}$ = 0.22), with pairwise comparisons (Bonferroni–Holm corrected) showing greater movement times for greater distances (*P*'s < 0.001, *d*'s > 2.70). In addition, the *effective* index of difficulty was calculated for each participant (49), and group averages were taken over participants (Fig. 6c, right panel). A mixed-effect model was fit, which showed a group-level intercept of 35 ms (*P* = 0.044) and a slope of 243 ms/bit (*P* < 0.001), comparable in magnitude to Pointing performance (40, 50) and exhibited the classic linear relationship between movement time and index of difficulty (48).

### Speed scales with distance

Speed profiles showed scaling with movement distance, where further movements showed higher velocities throughout (Fig. 6d, left panel) (45, 47). This can be assessed more directly by finding the peak speed reached on trials, which increased with movement distance (Fig. 6d, right panel). A repeated measures ANOVA on participant median peak speeds found a significant main effect of movement distance (*F*(1.17, 99.57) = 980.17, *P* < 0.001, $\eta_{G}^{2}$ = 0.56), with greater speeds at greater movement distances (*P*'s < 0.001, *d*'s > 2.66). Further, a repeated measures ANOVA on the within-subject variability of peak speed found a significant main effect of movement distance (*F*(1.18, 100.52) = 120.10, *P* < 0.001, $\eta_{G}^{2}$ = 0.28), with greater variability for greater movement distances (*P*'s < 0.001, *d*'s > 0.86), consistent with signal-dependent noise (51).

### Greater variability along the primary movement axis

To assess variability in feed-forward movement commands, we evaluated early kinematic markers, which have been shown to correlate well with end-point kinematics for uncorrected movements (52, 53). Cursor positions at the primary movement end were fit with 95% error ellipses per participant (Fig. 6e). A repeated measures ANOVA on participant ellipsoid aspect ratios found a significant main effect of movement distance (*F*(1.93, 164.03) = 52.26, *P* < 0.001, $\eta_{G}^{2}$ = 0.08). Aspect ratios were significantly greater than 1 for all distances (2.14–2.51, *P*'s < 0.001, *d*'s > 2.16), indicating more variability in extent than direction (52, 53).

### Systematic angle bias

The group showed a consistent bias in the angle of their movement at the end of the primary movement (54, 55). The average directional error appeared to follow a curve with two peaks and troughs (Fig. 6f), consistent with previous studies using arm reaches and mouse Pointing (55, 56), as well as studies in progress from our laboratory on mouse Pointing movements. A repeated measures ANOVA on participant median directional errors showed a main effect of movement angle (*F*(4.46, 334.29) = 25.16, *P* < 0.001, $\eta_{G}^{2}$ = 0.22).

### Concurrent improvements in temporal and spatial variables

**Fig. 7.**
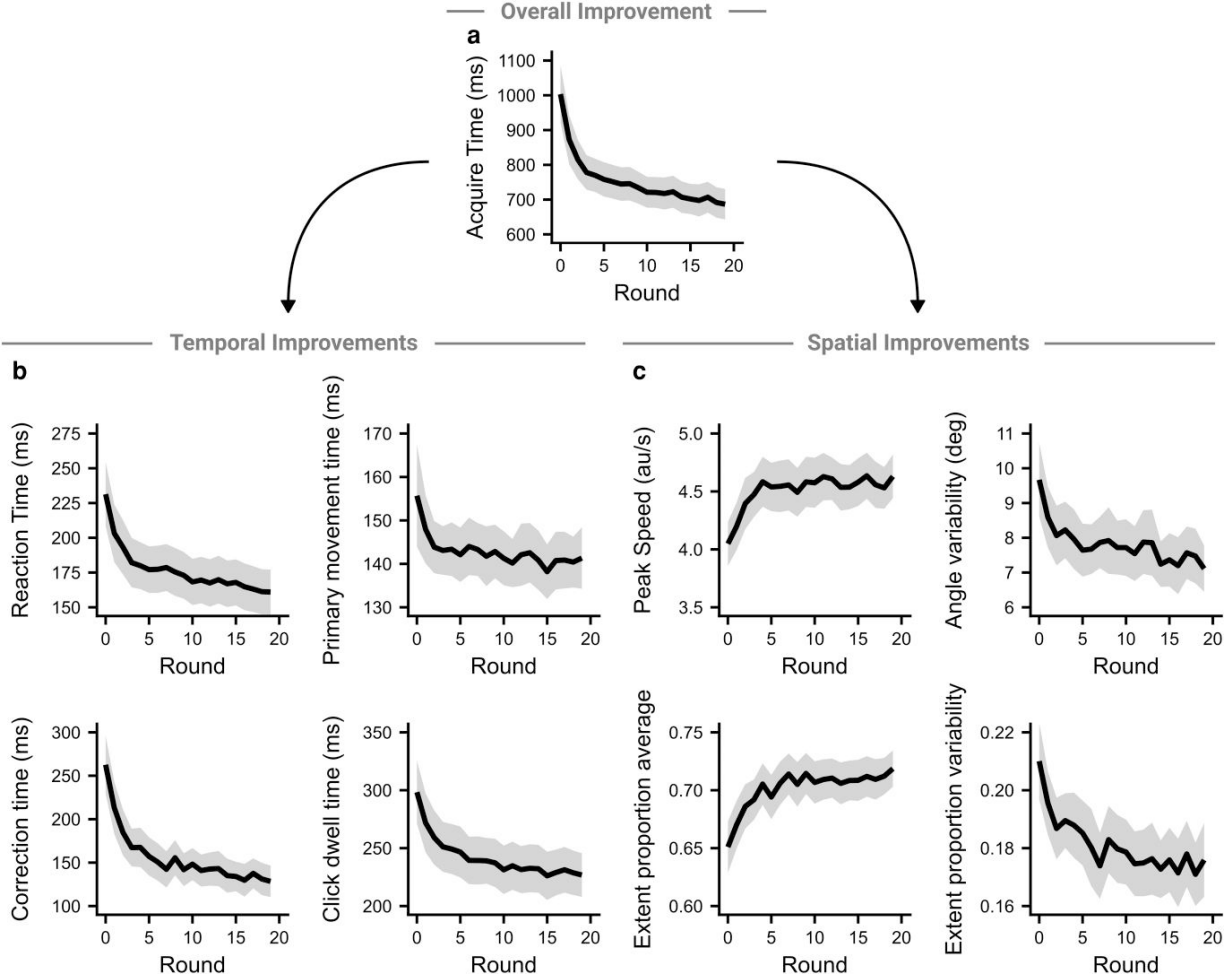
Temporal and spatial variables show concurrent improvements over the experiment. a) The main measure of performance on the task, the acquire time, improved continuously over the course of the experiment. The improvements appeared to consist of two phases—an initial rapid improvement followed by a sustained but slower improvement. b) The constituent temporal variables all show improvements over the experiment. c) The tested spatial variables also show improvements over the experiment. For all panels, thick line shows group mean and shaded regions show 95% CI.

In line with a previous study looking at effects of practice in an aim-training game (18), participants improved their performance over the course of the experiment (Fig. 7a), improving their acquire time by 317 ms over the 20 rounds ([249–385], *t*(85) = 9.17, *P* < 0.001, *d* = 0.99). Performance improved rapidly over the first five rounds, followed by a sustained slower improvement. The improvement in acquire time did not significantly differ by input device (*t*(84) = 1.82, *P* = 0.072, *d* = 0.40), gaming hours (*F*(4, 80) = 1.40, *P* = 0.242, $\eta_{G}^{2}$ = 0.07), or whether participants played FPS games (*t*(83) = −1.20, *P* = 0.235, *d* = 0.28). Participant performance by the end of the experiment did not depend on gaming hours (*F*(4, 80) = 1.55, *P* = 0.197, $\eta_{G}^{2}$ = 0.07), but mouse users were significantly faster than trackpad users (mouse: 614 ms; trackpad: 793 ms, *t*(84) = 4.31, *P* < 0.001, *d* = 0.95), and FPS players were significantly faster than non-FPS players (FPS players: 588 ms; non-FPS players: 727 ms, *t*(83) = 2.99, *P* = 0.004, *d* = 0.70).

As in the first experiment, we can divide the total acquire time into different phases of the movement (Fig. 7b). All phases of the movement showed significant improvements (reaction time: 70 ms [55–86 ms], *t*(85) = 9.03, *P* < 0.001, *d* = 0.97; primary movement: 14 ms [4–25 ms], *t*(85) = 2.67, *P* = 0.009, *d* = 0.29; corrections: 134 ms [105–164 ms], *t*(85) = 8.92, *P* < 0.001, *d* = 0.96; click dwell: 72 ms [51–92 ms], *t*(85) = 6.98, *P* < 0.001, *d* = 0.75). Given that each phase of the movement improved, we would expect this to be accompanied by improvements in the spatial variables. All four spatial variables showed significant improvements over the experiment (peak speed: 0.58 au/s [0.41–0.76 au/s], *t*(85) = 6.51, *P* < 0.001, *d* = 0.70; angle variability: 2.56° [1.60°–3.52°], *t*(85) = 5.25, *P* < 0.001, *d* = 0.57; extent average: 0.07 [0.05–0.09], *t*(85) = 6.68, *P* < 0.001, *d* = 0.72; extent variability: 0.03 [0.02–0.05], *t*(85) = 4.37, *P* < 0.001, *d* = 0.47). None of the improvements significantly differed between input device (*P*'s > 0.163, *d*'s < 0.31) other than click dwell time, which improved significantly more for trackpad users (mouse: 52 ms; trackpad: 100 ms, *t*(84) = 2.33, *P* = 0.022, *d* = 0.51).

### Spatial variables explain individual differences

To understand whether the spatial variables were important predictors of performance, we looked at whether the acquire time across the experiment was significantly predicted by any of the spatial variables used. A linear mixed effect model was fit, with acquire time as the dependent variable, the four spatial measures as independent variables, a random intercept for participants, and a random slope for round per participant. The model (marginal *R*^2^ = 0.22, conditional *R*^2^ = 0.89) showed that all four spatial variables significantly predicted acquire time. A 1 SD (scaled across the group) increase in the average and variability in the primary movement extent led to a 34 ms lower (*P* < 0.001) and 25 ms higher (*P* < 0.001) acquire time, respectively. The same increase for the average peak speed and variability in angle led to a 37 ms lower (*P* < 0.001) and 60 ms higher (*P* < 0.001) acquire time, respectively. All variables were significant predictors of acquire time for both mouse and trackpad users. That a small number of kinematic variables can predict task success highlights the utility in trying to understand the subcomponents of skilled FPS movements.

## Discussion

Despite widespread interest in the cognitive and perceptual abilities of video gamers, only recently have studies begun to assess the motor skills demonstrated. We isolated the skill of aiming at and shooting targets and analyzed its subcomponents using the kinematics of participant's movements. We found movements in an FPS-style Look context to be nearly identical to those in a more traditional Point context across a range of spatial and temporal measures, only differing slightly in reaction time, as well as correction time for trackpad users. We also showed that FPS movements are consistent with several classic observations from the reaching literature. We observed concurrent improvements in all tested spatial and temporal metrics with practice and found that the spatial metrics significantly predicted task performance. Our metrics can be generalized to studying FPS movements in other tasks, and actual gameplay data, providing an opportunity to advance our understanding of what factors set the best players apart.

### Studying skill in FPS games

Typical approaches to the study of skill involve abstract laboratory tasks or making restricted observations of real-world behavior using specialized equipment. Video games provide a unique opportunity to observe the development and maintenance of skill in its natural environment by simply recording the game inputs, typically provided by a keyboard and computer mouse. Only recently have studies begun to leverage this to assess skilled motor behavior in FPS games (17–21, 57, 58), though earlier studies have taken this approach to understand skill in real-time strategy games (14, 59).

We found that participants improved their Look performance with practice during the aim-training task (experiment 2), with an initial period of rapid improvement followed by a sustained but slower learning. This dual-rate pattern is consistent with improvements in hit speeds previously found (18) and with classic studies of motor learning (60–62). To move beyond broad measures of performance, we used the kinematics of participants’ movements to understand this development across a range of spatial and temporal metrics, finding that players improved across all metrics tested. Further, we characterized the importance of these measures to overall performance, finding all spatial metrics to be significant predictors of task performance. While mouse users acquired targets faster than trackpad users, we found that task improvements were similar across input devices, consistent with other work comparing motor learning between input devices (25).

While other studies have addressed similar questions and observed results consistent with our own, finding that similar temporal measures improved with practice (20) and reaction time and movement precision were significant correlates of motor skill (17), we believe our metrics have a number of advantages over those previously reported. Where Toth et al. (20) investigated an analogous measure to our click dwell time, it was based on when participants’ movement speed last dropped below a threshold. This allows the phase to begin before the target is reached, with subthreshold movements traversing the remaining distance. We instead delineated correction and click dwell phases by when the target was entered for the last time, ensuring that our click dwell metric only accrued time when a trial could have been ended successfully. Further, Donovan et al. (17) derived metrics from sigmoid fit to submovements, but this forces a model onto the data that assumes that submovements are distinct and ballistic; yet, individual trial speed profiles shown in Fig. 4e and other studies investigating speeded reaches with online feedback (63, 64) find asymmetric speed profiles and discrete corrections while speed was still high. Therefore, we used well-validated techniques to identify discontinuities in speed profiles (65, 66) to characterize spatial metrics at the end of the primary movement, making fewer assumptions about the exact form of the movements.

While the studies discussed thus far, and our own, utilized purposely simplified aim-trainer style tasks (to isolate specific components of behavior), the kinematic analyses described here are also applicable to FPS games generally. Indeed, similar analyses have been performed on actual gameplay data (21), finding professional players execute more effective shots, exhibit lower reaction times, and make more efficient movements. We believe that the utility of this approach will be most apparent if researchers can collect such gameplay data at scale to describe differences within a wide population of players. As the environmental constraints are weaker than those in an aim trainer (like nonstationary targets, multiple ideal aiming locations, and simultaneous character movement), the analysis techniques will need to account for the greater range of behaviors that may be observed.

Experiments using the FPS gaming approach described here could also have utility for studying individuals with movement disorders and younger participants. Assessment of how movement is impaired in conditions such as developmental coordination disorder and autism is typically made using standard reaching tasks (67, 68). We suggest that experiments modeled on FPS games, which allow detailed kinematic analysis of movements, might provide experiences more closely aligned with recreational activities and lead to higher degrees of engagement.

### FPS games inform our understanding of the neural control of movement

To understand whether there were any fundamental differences in how participants controlled their movements in the Look and Point contexts, we fully equated the movements required such that an identical mouse input would produce the same relative movement in the game. Participants required a 53 ms larger time limit to complete Looking movements, accounted for by larger reaction and correction times, each taking around 25 ms longer (though correction times were only elevated for trackpad users), while spatial features of the movements were nearly identical between contexts. A lack of familiarity with FPS games could not explain this effect, as the context difference did not significantly differ based on whether participants played them.

The only other study to compare these two contexts found Looking movements took around 230 ms longer than Pointing movements (40). This difference was quantified between the intercepts of linear regressions of movement time against index of difficulty, sometimes interpreted as measuring processes distinct from the movement itself (69), so it could be consistent with our finding of greater reaction times. However, Pointing was assessed via cursor movements to 2D rectangular targets and Looking in Unreal Tournament where users shot aliens, so several factors beyond the visual feedback difference could contribute, for example, if target localization was more demanding for the 3D task. Such confounds hamper interpretation of the observed differences in Looser et al. (40) but are addressed by our task design, which shows more modest context differences. This controlled comparison required a Looking context that is less rich in visual cues than a typical FPS game, however, so future work could investigate similar kinematic decomposition in more FPS-like environments.

Despite the two visual contexts entailing fundamentally different mappings between movement and visual motion feedback, behavior was highly similar. This is important because preeminent models of motor control propose that sensorimotor behavior is guided by a sensory state estimate generated through comparison of actual and predicted sensory feedback (32, 36, 70). Critically, if forward models predict primary sensory input, then feedback control would require different forward models for Point and Look contexts, as the sensory consequences of the same motor command evolve differently over time. It has previously been proposed that we maintain distinct models for different movement contexts to alleviate the complexity of a single controller accounting for all contextual information (71, 72).

It is unlikely that Look and Point contexts require entirely different internal models. Most of our participants (38 of the 50 in experiment 1) reported not playing FPS games, and 11 reported playing no video games at all. All were regular computer users, with a wealth of experience in the Point context. If distinct internal models were required for these contexts, the well-practiced Point movements should be relatively accurate and fast while Look movements would be slow and inaccurate, requiring de novo development of an internal model or association of arbitrary sensory feedback with existing motor commands. Visuomotor tasks that require such learning require an extended period of practice to approach good performance (62, 73–76). Instead, the majority of participants in our experiments could readily perform movements in the Look context, and performance was highly correlated across the two contexts.

Our results suggest that participants’ movements were largely impervious to the specifics of visual motion that result from movement. Instead, they appear to infer the relative positions of target and effector despite large differences in visual motion. These observations align with existing motor control theory if forward models in these frameworks make predictions of displacement vectors for visuospatial coordinates, allowing the motor system to use the same internal model for both movement contexts without a requirement to specify the categorical differences in visual motion (or more qualitative factors such as color). These complex facets of a visual scene would, however, need to be parsed upstream in higher visual areas. There is good evidence that the posterior parietal cortex combines hand and gaze reference frames into a hand-target displacement vector that is used in movement correction (77–79). Though speculative, we propose that visual target and effector positions for Look and Point would be parsed by the visual system at or before this step in the parietal cortex, and that forward model sensory predictions would be at the level of the combined hand-target displacement vector rather than any primary sensory modality. The ability of such a modular hierarchy to deal with stark differences in visual motion may help explain why movement vectors have been shown to be a critical link for both motor planning and learning (52, 80–82) and why visuomotor adaptation may occur at the step between parietal cortex and premotor cortices (83). We do wish to reiterate that these proposals are speculative.

In pointing movements, the eyes will typically saccade to the movement target before the hand movement is initiated and fixate it for the duration of the movement. Larger hand movement errors are made if the target is not foveated during these movements, suggesting this process improves target localization (84, 85). As both contexts appear identical before movement is initiated, we would expect a similar initial saccade for both Point and Look. However, upon moving the mouse, the target will begin to move in the Look context, which might require an extra saccade or smooth pursuit to maintain foveation during the final parts of the movement, with previous work suggesting the majority of fixations in FPS games are located around the aiming reticle (86). As the data were collected online, we could not collect detailed eye tracking data, so future work should compare patterns of eye movements between contexts and clarify whether they do differ, and if so, whether it can account for increased acquire times in the Look context.

### Wider applications

A perennial issue for FPS games is detecting cheats who gain an unfair advantage through use of third-party software that allows, for example, enemies to be seen through walls or automatically aimed at. Analyzing gameplay data for abnormal aiming abilities or other behavioral patterns has been suggested as an effective method for cheat detection (87). Our analysis of kinematics is both more general and precise than previous work and could be readily adapted to help detect cheaters.

As professional e-sports teams increasingly look to use analytical approaches to inform training programs (88), the analysis of kinematic metrics may allow tailored training, for instance, increasing focus on clicking predictively to reduce click dwell time. Further, players in teams are typically assigned a specific role to fulfill, like executing accurate shots with a sniper rifle, and a more thorough understanding of the strengths and weaknesses of the team roster may allow better role assignment.

## Methods

### Participants

Participants were recruited through the online testing platform Prolific and were paid £6 upon completion. They were only recruited if they resided in the United Kingdom or the United States, had English as a first language, and had a Prolific approval rating of 95% or above. Given the experiments were completed online, which is typically associated with noisier responses within and between participants compared with laboratory studies (e.g. Tsay et al. (25)), we applied stringent screening criteria to ensure the final sample was sufficiently high quality. The experiments were approved by the School of Psychology Ethics Committee at the University of Leeds, and participants gave informed consent via a web form prior to starting the study.

In experiment 1, 12 of the 62 participants were removed from analysis, giving a final sample of 50 participants (16 males, 34 females; mean age ± SD = 38 ± 13, age range = 21–66; 26 optical mouse users, 24 trackpad users). We removed five participants whose frame rate was either too low (<30 fps, as kinematic analysis resolution was poor for low frame rates) or differed between contexts (>10 fps, to remove frame rate–dependent changes in performance). A further three participants were excluded because their performance had not stabilized by the end of a block (success rate more than 10% away from intended 50% success rate over the last 40 trials of both staircases per context). Finally, four participants were excluded whose difference in time limit between the two contexts was above 3 median absolute deviations (MADs; calculated throughout using a consistency constant of 1.4826 to make it a robust estimator of SD) away from the group median difference (between 4.72 and 9.78, far higher than usual cutoffs), representing a subset of participants who struggled to perform in the Look context. Their performance was around 500–1,000 ms worse in the Look context, with greatly increased reaction and correction times. The latter appeared to be driven by shorter primary movements (∼0.3 au reduction), requiring greater feedback corrections, which introduced weak significant context differences for the primary movement extent and peak speed. While the results were otherwise the same at the group level with their inclusion and would not change our interpretation of the results, we chose to exclude these participants so that the group-level estimates of context differences were not unduly influenced by a small number of outliers.

In experiment 2, 14 of the 100 participants were removed from analysis, giving a final sample of 86 participants (44 males, 41 females; mean age ± SD = 43 ± 13, age range 22–78; 51 optical mouse users, 35 trackpad users). We removed 10 participants whose average frame rate was less than 30 fps or dropped by more than 10 fps between the first and last round, and a further four participants were excluded whose median acquire time was more than 3 MADs from the group median over the experiment (3.07–4.92). Exclusion of these participants does not change any statistical results.

### Apparatus

Participants used their own personal computer to complete the experiments and were restricted to users on a laptop or desktop using Prolific's screening tool. The experiments were created using the Unity game engine (2019.4.15f) and the Unity Experiment Framework (89) and delivered via a WebGL build hosted on a web page, with data uploaded to a remote database. Given participants used their own computers with varying sizes and aspect ratios, the physical size of the task was not consistent across participants but was developed to be visible on a 4:3 aspect ratio monitor. The height of the scene, 4 au, always took up the full height of the participant's monitor, with wider aspect ratios featuring more of a task-irrelevant background texture. Full screen was forced throughout, and during the experiment, the desktop cursor was hidden and locked so it could not be used to interact with the web page. Instead, the raw mouse or trackpad input was used to perform in-game movements of the cursor (eliminating mouse acceleration from the operating system). The sensitivity of in-game movements was initially calibrated to be similar to the participants’ desktop cursor, which could be adjusted in a calibration stage.

### Equating Pointing and Looking movements

See online supplementary material.

### Experimental task and procedure

In both experiments, participants first filled in details on their age, gender, whether they were using a mouse or trackpad, a desktop or laptop, and then pressed a button to ensure they could hear game audio. During this stage, participants used their usual desktop cursor to navigate the form, and the cursor movements in pixels and Unity game units were tracked to provide an initial calibration for the in-game cursor sensitivity that was around their regular desktop cursor sensitivity. Following this, the desktop cursor was hidden and locked, and interactions were only possible using their in-game cursor. Participants were shown a brief cutscene to provide exposition for the game (popping deadly nonsentient space bubbles), before progressing to a tutorial that introduced elements of a trial sequentially and interactively. At the end of the tutorial, participants were required to complete practice trials in each context, during which they could toggle context and adjust their cursor sensitivity. After completing at least 20 trials in each context, they could progress to the main task.

### Experiment 1

Participants used their computer mouse or trackpad to try to move to and shoot a target using an FPS-style cursor in either the Look or Point mode, depending on experimental condition. Participants saw a circular target plane (3 au diameter), on top of which targets could appear. The target plane was black, with a thin white ring around it. Behind this was a dark, space-themed background. A start point (0.1-au-diameter circle, initially colored orange) was located at the center of the target plane. Participants were required to move their in-game cursor, a small white circle surrounded by a thin white ring (diameter 0.15 au), to the start point and left-click to initiate a trial. If participants were within 0.05 au of the start point when making a homing movement, the cursor snapped to the start point to provide an FPS-style “auto-aim.” Upon left-clicking the start point, the color of the start point would turn green to indicate participants could move and a target (0.2-au-diameter magenta circle) would immediately appear on the target plane in one of eight locations. Potential targets were placed in increments of 45° around a virtual circle of radius 1 au, where 0° represented a target directly right of the center. Participants had to move to and click on the target within a time limit to shoot it, in which case it exploded and a shooting sound was made; otherwise, it would disappear and make a whooshing sound. A shot was only successful if, at the time of a left-click, the center of the cursor intercepted the target circle (there was no limit on the number of unsuccessful shots allowed within a trial). This feedback was provided for 300 ms, during which the start point was colored gray. After the feedback disappeared, the start point turned orange, indicating a new trial could begin.

Participants completed 640 experimental trials, split into a 320-trial block of Pointing and a 320-trial block of Looking. The order of the blocks was counterbalanced across participants, with half completing the Point block first. The blocks were arranged into cycles of eight trials, where each of the possible target angles was tested once in a random order. To induce the sort of time pressure experienced in a typical FPS game, the time limit within which targets had to be shot was continuously staircased throughout the experiment, using a one-up/one-down method. Within each block, two staircases were interleaved, one starting at a time limit of 300 ms and another starting at 1,500 ms, with the order shuffled such that the staircase switched after most trials. After a successful or unsuccessful trial, the time limit for the tested staircase was reduced or increased by 30 ms, respectively. By the end of each block, each staircase's time limit should become asymptotic at a value that gives roughly a 50% success rate. Participants were given a self-paced break every 80 trials. Between blocks, participants were informed the context would switch. Following completion of the experimental trials, participants completed a questionnaire probing their enjoyment of the game, perceived lag in the game, gaming experience, and any other comments. Video S1 demonstrates trials from this experiment.

### Experiment 2

Participants only completed movements in the Look context. Experimental trials began the same as in experiment 1, up until participants had clicked the start point. After the start point was clicked, it disappeared and a target appeared at one of three distances (0.4, 0.6, and 0.8 au) and eight angles (0°–315° in 45° increments). Simultaneously, the upcoming target after the current had been shot was shown as a hollow, faded magenta circle. This was implemented as pilot testing showed that without knowledge of the upcoming target, participants moved toward the workspace center following a successful shot, presumably to be equidistant from any given workspace area until the new target had been visually processed. Upon successfully shooting the current target, the upcoming target immediately became the new current target, and a new upcoming target was revealed. The new upcoming target was always located in a new location at one of the three distances and eight angles away from the current target. Participants continued shooting targets until 48 targets had been shot. While movements had no time limit, participants were told to complete each round as quickly as possible, with an on-screen timer visible during and after the round showing how long it had taken.

The 48 targets per round were arranged into two uninterrupted cycles, where each combination of the three distances and eight angles was tested once per cycle, with the criterion that no individual target could be located more than 1 au from the center of the workspace. The position of targets in each round was generated by simulating new sequences until this criterion was met. This had the effect of making seemingly random sequences of targets, while controlling the statistics of the reaches and staying within the intended workspace. Participants completed 20 rounds of 48 movements and were given a self-paced break between each round. Following completion of the rounds, participants completed a questionnaire probing game enjoyment, perceived lag, strategies they used to improve, gaming experiment, and any other comments, with many participants indicating they had enjoyed playing this experiment. Video S2 demonstrates a round from this experiment.

### Data and statistical analyses

See online supplementary materials.

## Supplementary Material

pgad249_Supplementary_Data

## Data Availability

All data and scripts to analyze the data are available at https://osf.io/xd8sm/.

## References

[1] Tristão H . 2022. Esports audience will pass half a billion in 2022 | Esports market analysis. Newzoo. (September 6, 2022).

[2] Bediou B , et al 2018. Meta-analysis of action video game impact on perceptual, attentional, and cognitive skills. Psychol Bull. 144:77–110. 29172564 10.1037/bul0000130

[3] Green CS , Bavelier D. 2012. Learning, attentional control, and action video games. Curr Biol. 22:R197–R206. 22440805 10.1016/j.cub.2012.02.012 PMC3461277

[4] Pedraza-Ramirez I , Musculus L, Raab M, Laborde S. 2020. Setting the scientific stage for esports psychology: a systematic review. Int Rev Sport Exerc Psychol. 13:319–352.

[5] Campbell MJ , Toth AJ, Moran AP, Kowal M, Exton C. 2018. eSports: A new window on neurocognitive expertise? In: Marcona S, Sarkar M, editors, Sport and the brain: The science of preparing, enduring, and winning, part C (Vol. 240). Cambridge (MA): Academic Press. p. 161–174. 10.1016/bs.pbr.2018.09.006 30390829

[6] Reeves S , Brown B, Laurier E. 2009. Experts at play: understanding skilled expertise. Games Cult. 4:205–227.

[7] Pickavance JP , et al 2022. Sensorimotor ability and inhibitory control independently predict attainment in mathematics in children and adolescents. J Neurophysiol. 127:1026–1039. 35196148 10.1152/jn.00365.2021

[8] Coll S-M , Foster NEV, Meilleur A, Brambati SM, Hyde KL. 2020. Sensorimotor skills in autism spectrum disorder: a meta-analysis. Res Autism Spectr Disord. 76:101570.

[9] Rodriguez MC , et al 2019. Emotional and behavioral problems in 4- and 5-year old children with and without motor delays. Front Pediatr. 7:474. 31803697 10.3389/fped.2019.00474 PMC6877720

[10] Mané A , Donchin E. 1989. The space fortress game. Acta Psychol (Amst.). 71:17–22.

[11] Murphy S , Games V. 2009. Competition and exercise: a new opportunity for sport psychologists? Sport Psychol. 23:487–503.

[12] Stafford T , Dewar M. 2014. Tracing the trajectory of skill learning with a very large sample of online game players. Psychol Sci. 25:511–518. 24379154 10.1177/0956797613511466

[13] Aung M , et al 2018. Predicting skill learning in a large, longitudinal MOBA dataset. In: 2018 IEEE Conference on Computational Intelligence and Games (CIG). IEEE. p. 1–7.

[14] Huang J , Yan E, Cheung G, Nagappan N, Zimmermann T. 2017. Master maker: understanding gaming skill through practice and habit from gameplay behavior. Top Cogn Sci. 9:437–466. 28198102 10.1111/tops.12251

[15] Stafford T , Devlin S, Sifa R, Drachen A. 2017. Exploration and skill acquisition in a major online game. In: The 39^th^ Annual Meeting of the Cognitive Science Society, p. 7.

[16] Sapienza A , Zeng Y, Bessi A, Lerman K, Ferrara E. 2018. Individual performance in team-based online games. R Soc Open Sci. 5:180329–180314. 30110428 10.1098/rsos.180329 PMC6030337

[17] Donovan I , et al 2022. Assessment of human expertise and movement kinematics in first-person shooter games. Front Hum Neurosci. 16:979293. 36523441 10.3389/fnhum.2022.979293 PMC9744923

[18] Listman JB , Tsay JS, Kim HE, Mackey WE, Heeger DJ. 2021. Long-term motor learning in the “wild” with high volume video game data. Front Hum Neurosci. 15:777779. 34987368 10.3389/fnhum.2021.777779 PMC8720934

[19] Toth AJ , Ramsbottom N, Constantin C, Milliet A, Campbell MJ. 2021. The effect of expertise, training and neurostimulation on sensory-motor skill in esports. Comput Hum Behav. 121:106782.

[20] Toth AJ , Hojaji F, Campbell MJ. 2023. Exploring the mechanisms of target acquisition performance in esports: the role of component kinematic phases on a first person shooter motor skill. Comput Hum Behav. 139:107554.

[21] Park E , et al 2021. Secrets of Gosu: understanding physical combat skills of professional players in first-person shooters. In: Proceedings of the 2021 CHI Conference on Human Factors in Computing Systems. ACM. p. 1–14.

[22] Coltman SK , van Beers RJ, Medendorp WP, Gribble PL. 2021. Sensitivity to error during visuomotor adaptation is similarly modulated by abrupt, gradual, and random perturbation schedules. J Neurophysiol. 126:934–945. 34379553 10.1152/jn.00269.2021

[23] Kim OA , Forrence AD, McDougle SD. 2022. Motor learning without movement. *Proceedings of the National Academy of Sciences* 119(30):e2204379119. doi:10.1073/pnas.2204379119 PMC9335319 35858450

[24] Smeets J , Brenner E. 2003. Fast corrections of movements with a computer mouse. Spat Vis. 16:365–376. 12858957 10.1163/156856803322467581

[25] Tsay JS , Ivry RB, Lee A, Avraham G. 2021. Moving outside the lab: the viability of conducting sensorimotor learning studies online. Neurons Behav Data Anal Theory. 5:1–22.

[26] MacKenzie IS , Kauppinen T, Silfverberg M. 2001. Accuracy measures for evaluating computer pointing devices. In: Proceedings of the SIGCHI Conference on Human Factors in Computing Systems—CHI ’01. ACM Press. p. 9–16.

[27] Mithal AK , Douglas SA. 1996. Differences in movement microstructure of the mouse and the finger-controlled isometric joystick. In: Proceedings of the SIGCHI Conference on Human Factors in Computing Systems Common Ground—CHI ’96. ACM Press. p. 300–307.

[28] Phillips JG , Triggs TJ. 2001. Characteristics of cursor trajectories controlled by the computer mouse. Ergonomics 44:527–536. 11345495 10.1080/00140130121560

[29] Walker N , Meyer DE, Smelcer JB. 1993. Spatial and temporal characteristics of rapid cursor-positioning movements with electromechanical mice in human-computer interaction. Hum Factors. 35:431–458. 8244410 10.1177/001872089303500304

[30] Desmurget M , Grafton S. 2000. Forward modeling allows feedback control for fast reaching movements. Trends Cogn Sci. 4:423–431. 11058820 10.1016/s1364-6613(00)01537-0

[31] Raibert MH . 1978. A model for sensorimotor control and learning. Biol Cybern. 29:29–36. 656477 10.1007/BF00365233

[32] Diedrichsen J , Shadmehr R, Ivry RB. 2010. The coordination of movement: optimal feedback control and beyond. Trends Cogn Sci. 14:31–39. 20005767 10.1016/j.tics.2009.11.004 PMC4350769

[33] Todorov E . 2004. Optimality principles in sensorimotor control. Nat Neurosci. 7:907–915. 15332089 10.1038/nn1309 PMC1488877

[34] Kawato M . 1999. Internal models for motor control and trajectory planning. Curr Opin Neurobiol. 9:718–727. 10607637 10.1016/s0959-4388(99)00028-8

[35] Miall RC , Wolpert DM. 1996. Forward models for physiological motor control. Neural Netw. 9:1265–1279. 12662535 10.1016/s0893-6080(96)00035-4

[36] Scott SH . 2012. The computational and neural basis of voluntary motor control and planning. Trends Cogn Sci. 16:541–549. 23031541 10.1016/j.tics.2012.09.008

[37] Shadmehr R , Krakauer JW. 2008. A computational neuroanatomy for motor control. Exp Brain Res. 185:359–381. 18251019 10.1007/s00221-008-1280-5 PMC2553854

[38] Wolpert DM , Miall RC, Kawato M. 1998. Internal models in the cerebellum. Trends Cogn Sci. 2:338–347. 21227230 10.1016/s1364-6613(98)01221-2

[39] Medendorp WP , Heed T. 2019. State estimation in posterior parietal cortex: distinct poles of environmental and bodily states. Prog Neurobiol. 183:101691. 31499087 10.1016/j.pneurobio.2019.101691

[40] Looser J , Cockburn A, Savage J. 2005. On the validity of using first-person shooters for Fitts’ law studies. People Comput XIX. 2:33–36.

[41] Elliott D , et al 2010. Goal-directed aiming: two components but multiple processes. Psychol Bull. 136:1023–1044. 20822209 10.1037/a0020958

[42] Abend W , Bizzi E, Morasso P. 1982. Human arm trajectory formation. Brain J Neurol. 105:331–348. 10.1093/brain/105.2.331 7082993

[43] Morasso P . 1981. Spatial control of arm movements. Exp Brain Res. 42:223–227. 7262217 10.1007/BF00236911

[44] Sergio LE , Scott SH. 1998. Hand and joint paths during reaching movements with and without vision. Exp Brain Res. 122:157–164. 9776514 10.1007/s002210050503

[45] Atkeson C , Hollerbach J. 1985. Kinematic features of unrestrained vertical arm movements. J Neurosci. 5:2318–2330. 4031998 10.1523/JNEUROSCI.05-09-02318.1985 PMC6565321

[46] Kistemaker DA , Wong JD, Gribble PL. 2014. The cost of moving optimally: kinematic path selection. J Neurophysiol. 112:1815–1824. 24944215 10.1152/jn.00291.2014 PMC4200004

[47] Brown JS , Slater-Hammel AT. 1949. Discrete movements in the horizontal plane as a function of their length and direction. J Exp Psychol. 39:84. 18124068 10.1037/h0062478

[48] Fitts PM . 1954. The information capacity of the human motor system in controlling the amplitude of movement. J Exp Psychol. 47:381. 13174710

[49] Soukoreff RW , MacKenzie IS. 2004. Towards a standard for pointing device evaluation, perspectives on 27 years of Fitts’ law research in HCI. Int J Hum-Comput Stud. 61:751–789.

[50] MacKenzie IS , Ware C. 1993. Lag as a determinant of human performance in interactive systems. In: Proceedings of the SIGCHI Conference on Human Factors in Computing Systems—CHI ’93. ACM Press. p. 488–493.

[51] Harris CM , Wolpert DM. 1998. Signal-dependent noise determines motor planning. Nature 394:780–784. 9723616 10.1038/29528

[52] Gordon J , Ghilardi MF, Ghez C. 1994. Accuracy of planar reaching movements: I. Independence of direction and extent variability. Exp Brain Res. 99:97–111. 7925800 10.1007/BF00241415

[53] Messier J , Kalaska JF. 1999. Comparison of variability of initial kinematics and endpoints of reaching movements. Exp Brain Res. 125:139–152. 10204767 10.1007/s002210050669

[54] Begbie GH . 1959. Accuracy of aiming in linear hand-movements. Q J Exp Psychol. 11:65–75.

[55] Ghilardi MF , Gordon J, Ghez C. 1995. Learning a visuomotor transformation in a local area of work space produces directional biases in other areas. J Neurophysiol. 73:2535–2539. 7666158 10.1152/jn.1995.73.6.2535

[56] Chandy A , et al 2021. Motor biases are persistent and consistent.

[57] Boudaoud B , Spjut J, Kim J. 2022. Mouse sensitivity in first-person targeting tasks. In: 2022 IEEE Conference on Games (CoG). IEEE. p. 183–190.

[58] Boudaoud B , Spjut J, Kim J. 2022. FirstPersonScience: An Open Source Tool for Studying FPS Esports Aiming in Special Interest Group on Computer Graphics and Interactive Techniques Conference Talks. ACM. p. 1–2.

[59] Thompson JJ , McColeman CM, Stepanova ER, Blair MR. 2017. Using video game telemetry data to research motor chunking, action latencies, and complex cognitive-motor skill learning. Top Cogn Sci. 9:467–484. 28176483 10.1111/tops.12254

[60] Adams JA . 1952. Warm-up decrement in performance on the pursuit-rotor. Am J Psychol. 65:404. 12976564

[61] Smith MA , Ghazizadeh A, Shadmehr R. 2006. Interacting adaptive processes with different timescales underlie short-term motor learning. PLoS Biol. 4:e179. 16700627 10.1371/journal.pbio.0040179 PMC1463025

[62] Snoddy GS . 1926. Learning and stability. J Appl Psychol. 10:1–36.

[63] Fishbach A , Roy SA, Bastianen C, Miller LE, Houk JC. 2007. Deciding when and how to correct a movement: discrete submovements as a decision making process. Exp Brain Res. 177:45–63. 16944111 10.1007/s00221-006-0652-y

[64] Milner TE , Ijaz MM. 1990. The effect of accuracy constraints on three-dimensional movement kinematics. Neuroscience 35:365–374. 2381512 10.1016/0306-4522(90)90090-q

[65] Abrams RA , Pratt J. 1993. Rapid aimed limb movements: differential effects of practice on component submovements. J Mot Behav. 25:288–298. 15064195 10.1080/00222895.1993.9941650

[66] Meyer DE , Abrams RA, Kornblum S, Wright CE, Keith Smith J. 1988. Optimality in human motor performance: ideal control of rapid aimed movements. Psychol Rev. 95:340. 3406245 10.1037/0033-295x.95.3.340

[67] Foster NC , et al 2020. Getting off to a shaky start: specificity in planning and feedforward control during sensorimotor learning in autism spectrum disorder. Autism Res. 13:423–435. 31661192 10.1002/aur.2214

[68] Hyde C , Wilson PH. 2011. Dissecting online control in developmental coordination disorder: a kinematic analysis of double-step reaching. Brain Cogn. 75:232–241. 21256656 10.1016/j.bandc.2010.12.004

[69] Zhai S . 2004. Characterizing computer input with Fitts’ law parameters—the information and non-information aspects of pointing. Int J Hum-Comput Stud. 61:791–809.

[70] McNamee D , Wolpert DM. 2019. Internal models in biological control. Annu Rev Control Robot Auton Syst. 2:339–364. 31106294 10.1146/annurev-control-060117-105206 PMC6520231

[71] Haruno M , Wolpert DM, Kawato M. 2001. MOSAIC model for sensorimotor learning and control. Neural Comput. 13:2201–2220. 11570996 10.1162/089976601750541778

[72] Wolpert DM , Kawato M. 1998. Multiple paired forward and inverse models for motor control. Neural Netw. 11:1317–1329. 12662752 10.1016/s0893-6080(98)00066-5

[73] Lillicrap TP , et al 2013. Adapting to inversion of the visual field: a new twist on an old problem. Exp Brain Res. 228:327–339. 23700129 10.1007/s00221-013-3565-6

[74] Telgen S , Parvin D, Diedrichsen J. 2014. Mirror reversal and visual rotation are learned and consolidated via separate mechanisms: recalibrating or learning *de novo* ? J Neurosci. 34:13768–13779. 25297103 10.1523/JNEUROSCI.5306-13.2014 PMC6608381

[75] Wilterson SA , Taylor JA. 2021. Implicit visuomotor adaptation remains limited after several days of training. eneuro 8:ENEURO.0312-20.2021. 10.1523/ENEURO.0312-20.2021 PMC8362683 34301722

[76] Yang CS , Cowan NJ, Haith AM. 2021. De novo learning versus adaptation of continuous control in a manual tracking task. eLife 10:e62578. 34169838 10.7554/eLife.62578 PMC8266385

[77] Buneo CA , Jarvis MR, Batista AP, Andersen RA. 2002. Direct visuomotor transformations for reaching. Nature 416:632–636. 11948351 10.1038/416632a

[78] Buneo CA , Andersen RA. 2006. The posterior parietal cortex: sensorimotor interface for the planning and online control of visually guided movements. Neuropsychologia 44:2594–2606. 16300804 10.1016/j.neuropsychologia.2005.10.011

[79] Desmurget M , et al 1999. Role of the posterior parietal cortex in updating reaching movements to a visual target. Nat Neurosci. 2:563–567. 10448222 10.1038/9219

[80] Krakauer JW , Pine ZM, Ghilardi M-F, Ghez C. 2000. Learning of visuomotor transformations for vectorial planning of reaching trajectories. J Neurosci. 20:8916–8924. 11102502 10.1523/JNEUROSCI.20-23-08916.2000 PMC6773094

[81] Wang J , Sainburg RL. 2005. Adaptation to visuomotor rotations remaps movement vectors, not final positions. J Neurosci. 25:4024–4030. 15843604 10.1523/JNEUROSCI.5000-04.2005 PMC6724955

[82] Wu HG , Smith MA. 2013. The generalization of visuomotor learning to untrained movements and movement sequences based on movement vector and goal location remapping. J Neurosci. 33:10772–10789. 23804099 10.1523/JNEUROSCI.3761-12.2013 PMC4469867

[83] Tanaka H , Sejnowski TJ, Krakauer JW. 2009. Adaptation to visuomotor rotation through interaction between posterior parietal and motor cortical areas. J Neurophysiol. 102:2921–2932. 19741098 10.1152/jn.90834.2008 PMC2777823

[84] Neggers SFW , Bekkering H. 2000. Ocular gaze is anchored to the target of an ongoing pointing movement. J Neurophysiol. 83:639–651. 10669480 10.1152/jn.2000.83.2.639

[85] Prablanc C , Echallier JF, Komilis E, Jeannerod M. 1979. Optimal response of eye and hand motor systems in pointing at a visual target. Biol Cybern. 35:113–124. 518932 10.1007/BF00337436

[86] Kenny A , Koesling H, Delaney D, McLoone S, Ward T. 2005. A preliminary investigation into eye gaze data in a first person shooter game. In: Proceedings of the 19^th^ European Conference on Modelling and Simulation, p. 6.

[87] Pao H-K , Chen K-T, Chang H-C. 2010. Game bot detection via avatar trajectory analysis. IEEE Trans Comput Intell AI Games. 2:162–175.

[88] Waananen K . 2022. Touring Team Liquid and Alienware's The Pro Lab. Esports Insid. (November 21, 2022).

[89] Brookes J , Warburton M, Alghadier M, Mon-Williams M, Mushtaq F. 2019. Studying human behavior with virtual reality: the Unity Experiment Framework. Behav Res Methods. 52:455–463. 10.3758/s13428-019-01242-0 PMC7148262 31012061

