# Supplementary material for: Kinematic markers of skill in first-person shooter video games
Source: PNAS Nexus. 2023 Jul 31;2(8):pgad249. doi: 10.1093/pnasnexus/pgad249 (PMC10411933; doi:10.1093/pnasnexus/pgad249)
Supplement: pgad249_Supplementary_Data [file pgad249_supplementary_data.zip › PNASNEXUS-PNASNEXUS-2023-00487-T-s01.pdf]

Supporting information for:

Kinematic markers of skill in first-person shooter video games

Matthew Warburton, Carlo Campagnoli, Mark Mon-Williams, Faisal Mushtaq, J. Ryan Morehead

Corresponding author: Matthew Warburton and J. Ryan Morehead

Email: pscmwa@leeds.ac.uk and j.r.morehead@leeds.ac.uk

This PDF includes:

Supporting text

SI References

Other supplementary materials for this manuscript include the following:

Videos S1 to S2

Methods

Equating pointing and looking movements

Participants used their mouse or trackpad to manipulate the in-game cursor position in either the Point or Look contexts to attempt to 'pop' targets. A key requirement for the experiments were that a particular target could be reached in either context using an identical mouse input. For the Point context, the experiment is set up so that a participant's view of the scene is provided by a static orthographic camera. The raw mouse input on a given frame, provided as a difference in mouse position from the previous frame, is multiplied by the participant's calibrated sensitivity and added to the current cursor position. This setup mimics a typical 2D reaching task.

For the Look context, the raw mouse input is instead used to rotate an orthographic camera about a pivot, while the cursor is fixed central to the camera's view. For a given translation of the cursor from the middle of the workspace along a single axis in the Point context, o, this can be formulated as the opposite length of a right-angle triangle, which using a basic trigonometric identity gives $o = a \times\ tan(\theta)$, where a is the distance between the rotation pivot and the plane on which the cursor should translate and θ is the required rotation. For small rotations, a small-angle approximation holds where $\tan(\theta) \approx \theta$ and therefore $\theta \propto o$. The task was developed to have a maximum workspace of 1au in any direction from the centre, and a distance of 50au between the camera's rotation pivot and the target plane was used to ensure only small rotations were required. We then simply need to multiply the input by a coefficient, c, to get a rotation that approximates the equivalent Point translation. Here we pick a coefficient that minimises error at the target distance, $c = arctan\left(\frac{1}{50}\right) \approx 1.1457\ deg$. Over the intended workspace, this gave a maximum difference between the cursor's position on the target plane in the Point and Look modes of 5.13×10^{-5}au, which equates to a sub-pixel difference on a 1080p monitor. The raw mouse input on every frame was therefore multiplied both by the participant's calibrated sensitivity and this rotation coefficient, and added to the camera's current rotation. This provides a general framework for building experiments where traditional mouse Pointing or FPS-style mouse Looking can be used and compared in the same task.

Data analysis

Data were processed using R (version 4.2.2). The cursor position time series data, sampled at the participant's refresh rate by Unity, was resampled to a standard frequency of 100Hz using linear interpolation. This resampled data was then filtered using a second order, low-pass Butterworth filter with a 15Hz cut-off in the forward and reverse directions to give zero lag, with the start and end of each

trial’s time series temporarily padded to remove transient effects of the filter. Movements were segmented in Experiment 1 by extracting the period between the start-point being clicked and an outcome being registered, and in Experiment 2 by extracting the period between the previous and current target being clicked. Speed, acceleration, and jerk in the radial direction were obtained by numerically differentiating the position data in polar coordinates (using *pracma’s* gradient function), centred on the cursor position at the start of each movement segment. To visualise hand paths, movements in cartesian coordinates were resampled into 100 evenly spaced points between the start and end of the movement segment. Participant averages were produced as the mean position at each interpolated time step, and group averages found by averaging over all participants. The hand paths were used to derive spatial and temporal metrics, with within-subject averages calculated using medians to account for the typically skewed distributions of temporal measures.

Staircasing metric

In Experiment 1, the continuous staircasing led to participants being successful on roughly half the trials by the end of a block. We extracted each participant’s median time limit over the last 40 trials of each staircase per block to quantify the time required to be 50% likely to execute a successful shot, as this region appeared to contain roughly asymptotic behaviour.

Spatial metrics

Four spatial metrics were focussed on through both experiments, similar to metrics previously used (1). The progression of each measure could be visualised across a trial, by aligning movements at motion onset and calculating the relevant measure per time-step. However, this method produces profiles that do not necessarily reflect the individual trials well (e.g. in Figure 4e, the average speed profile looks smooth whereas many individual trials show secondary speed peaks), so measures were also extracted at a single, theoretically meaningful point in time. In Experiment 1, participant summary measures were calculated over successful trials from the last 40 observations per staircase in each context to ensure a consistent sample, as unsuccessful trials may be missing some movement features. In Experiment 2, measures were calculated over all movements within each round, giving one observation of each measure per round per participant.

Average Peak speed. The peak speed was the maximum radial speed reached on the trial, and medians were taken over these values to find the average peak speed reached.

Average extent at the end of the primary movement. For each time step, the extent in the direction of the current target was found by comparing the current cursor position to the straight line joining the cursor position at the start of the movement and the centre of the current target (ideal path). The current distance from the movement start point, and the angle from the ideal path, could then be used to find the extent in the direction of the target using $r \times \cos(\theta)$. This could be converted to a proportion by dividing by the length of the straight line between cursor start point and target centre, which was useful for Experiment 2 to collapse across movement distances.

We were particularly interested in the average extent at the end of the primary movement, as this is purported to represent where discrete feedback corrections begin (2), and hence should determine the magnitude of the feedback correction required. To identify the end of a primary movement, we followed a similar procedure to other papers (3, 4). Following the peak radial speed, we looked for any timestamps where (a) the radial speed fell below the movement threshold, indicating the movement either terminated or was about to reverse direction, (b) the radial acceleration crossed from negative to positive, indicating participants had sped up, or (c) the radial jerk crossed from positive to negative, indicating participants were 'braking'. The proportion of the extent covered was then found at the timestamp where the primary movement ended, and the median was taken to find the average in this value.

Variability in extent at the end of the primary movement. The variability was found by calculating the MAD of the proportion of the extent covered at the end of the primary movement.

Variability in angle at the end of the primary movement. We also extracted the hand angle, the difference in angle between straight lines connecting the starting point of the movement to either the target (ideal path) and the current cursor position. Positive hand angles indicated counter-clockwise errors. The MAD was calculated over hand angles at the end of the primary movement.

Temporal metrics

The kinematics were also used to establish temporal variables that sum to the total time required to shoot a given target, the acquire time. Reaction time was calculated first, here defined as the difference in time between a current target being shown and the first time where radial speed rose above 0.5au/s. The remaining period of the trial could be split up into further phases. The time between motion starting and the primary movement ending gives the primary movement time, and the time between the cursor entering the target and a successful click being registered gives the click dwell time. If the end of a primary movement occurred after the target had been entered, it was shortened to end

when the target had been entered, to make the primary movement and click dwell phases mutually exclusive. The remaining portion, between the primary movement ending and the target being entered, can then be treated as the correction time, the time required to execute discrete corrections to bring the cursor within the target. These phases are marked on a single example trial in Figure 5b. Average metrics were produced per participant by taking medians over the successful trials from the last 40 observations of each staircase per context in Experiment 1, and over all movements per round in Experiment 2.

Classic metrics

In Experiment 2, we also compared the movements to classic observations from reaching tasks, where additional metrics needed calculating. To understand movement time scaling with distance, the median movement time was calculated per distance, and also compared across effective index of difficulty, calculated following standardised methods (5). Effective index of difficulty was calculated as $ID = \log_2(1 + D_e/W_e)$, where D_e is the average straight-line distance between movement start and end points, and $W_e = 4.133\sigma$, where σ was the standard deviation of the end-points along the ideal direction of movement. This was calculated per nominal target distance per participant. Peak speed scaling was assessed by averaging the speed profiles or peak radial speed per target distance, or calculating the MAD of peak speed. Aiming biases were found by calculating the median hand angle from the ideal path per target angle, after hand angles outside 3 MADs from the group median were removed. The comparison of variability in extent and direction was performed by aligning movements at their start-point, rotating movements as if directed to a 90° target, and isolating the cursor position at the end of the primary movement. As with the bias analysis, end-points with a hand angle greater than 3 MADs from the group median were removed to ignore movements that were not target-directed. Error ellipsoids were fit per target distance per participant following previous studies (6, 7), giving the angles of the two principal axes and the variability along each axis. The aspect ratio here was calculated as the ratio of standard deviations of the axes aligned more closely to the extent and angle axes respectively. An aspect ratio > 1 indicated movements had more variability in extent than direction. The group average ellipsoid was found by averaging the centre, axis angles and standard deviations over participants.

Gaming experience

In both experiments, participants reported in the post-experiment questionnaire how many hours they played video games per week (options: I don't play video games, Up to 5 hours, Up to 10 hours, Up to 20 hours, More than 20 hours), and what video game genres they played from a checkbox list (with First-person shooter games as an option).

Statistical Analysis

Statistical analysis was performed using R (version 4.2.2). In Experiment 1, comparisons between contexts were made by performing Pearson correlations between metrics in the Look and Point context, with paired-samples t-tests used to assess average within-subject differences between contexts. These were both compared across input device, comparing correlation coefficients using Fisher's z-transform (*cocor* package), and comparing context differences using independent samples t-tests. Comparisons were also made between all metrics and the asymptotic time limit using Pearson correlations, to understand which might be important predictors of skill on the task. In Experiment 2, improvements in metrics over the experiment was assessed using paired-samples t-tests between the first and last rounds of the experiment, and again compared across input device using independent samples t-tests. Assessment of the metrics important for overall task performance was done by entering them into a linear mixed-effect regression (packages: *lme4*, *lmerTest* for Satterthwaite's approximation of p-values, and *MuMIn* for R^2 values) where acquire time was predicted by the four spatial metrics, each z-scored across the group, with a random intercept per participant and a random slope for the round per participant.

A variety of methods were utilised to compare the Look movements to classic findings. Comparisons across distance (movement time scaling, peak speed scaling, and aspect ratio of the error ellipsoids) or angle (bias across target angles) were done using a repeated-measures ANOVA (*afex* package, with Greenhouse-Geisser corrections) and followed up using one-sample or pairwise comparisons on the estimated marginal means (*emmeans* package) with Bonferroni-Holm corrections. The regression of effective index of difficulty on movement time was done using mixed-effect linear regression, with a fixed effect of effective index of difficulty, a random intercept per participant, and a random slope of effective index of difficulty per participant. Effect sizes for pairwise comparisons are reported using Cohen's d (*effectsize* package) and for ANOVA using generalised eta-squared (η_G^2). The statistical significance threshold was set at $p < .05$ throughout.

Video S1 (separate file): Video demonstrating the Pointing and Looking contexts for in Experiment 1. A label is overlaid indicating which context the video is currently demonstrating. Participants click a start-point in the middle of the circular target plane to start a trial. Participants have to move to and click the target within a time-limit, otherwise it will disappear. A successful shot will explode the target.

Video S2 (separate file): Video demonstrating an experimental round in Experiment 2. Participants start a round by clicking the start-point in the middle of the circular target plane. After this, a solid target shows the current target and a hollow one shows the following target. Upon a successful click to the current target, the following target becomes the current one and a new following target is revealed. Participants have to shoot 48 targets, arranged in seemingly random configurations, as quickly as they can.

SI References

1. I. Donovan, *et al.*, Assessment of human expertise and movement kinematics in first-person shooter games. *Front. Hum. Neurosci.* **16** (2022).
2. D. Elliott, *et al.*, Goal-directed aiming: Two components but multiple processes. *Psychol. Bull.* **136**, 1023–1044 (2010).
3. R. A. Abrams, J. Pratt, Rapid Aimed Limb Movements: Differential Effects of Practice on Component Submovements. *J. Mot. Behav.* **25**, 288–298 (1993).
4. D. E. Meyer, R. A. Abrams, S. Kornblum, C. E. Wright, J. Keith Smith, Optimality in human motor performance: ideal control of rapid aimed movements. *Psychol. Rev.* **95**, 340 (1988).
5. R. W. Soukoreff, I. S. MacKenzie, Towards a standard for pointing device evaluation, perspectives on 27 years of Fitts' law research in HCI. *Int. J. Hum.-Comput. Stud.* **61**, 751–789 (2004).
6. J. Gordon, M. F. Ghilardi, C. Ghez, Accuracy of planar reaching movements: I. Independence of direction and extent variability. *Exp. Brain Res.* **99**, 97–111 (1994).
7. J. Messier, J. F. Kalaska, Comparison of variability of initial kinematics and endpoints of reaching movements. *Exp. Brain Res.* **125**, 139–152 (1999).
